# Synchronous Occurrence of Chronic Myeloid Leukemia and Mantle Cell Lymphoma

**DOI:** 10.1155/2017/7815095

**Published:** 2017-02-08

**Authors:** Prajwol Pathak, Ying Li, Brian Allen Gray, William Stratford May, Merry Jennifer Markham

**Affiliations:** ^1^Division of Hematology and Oncology, Department of Medicine, University of Florida, Gainesville, FL, USA; ^2^Department of Pathology, University of Florida, Gainesville, FL, USA

## Abstract

Chronic myeloid leukemia (CML) and mantle cell lymphoma (MCL) are hematologic malignancies that originate from different oligopotent progenitor stem cells, namely, common myeloid and lymphoid progenitor cells, respectively. Although blastic transformation of CML can occur in the lymphoid lineage and CML has been related to non-Hodgkin lymphoma on transformation, to our knowledge, de novo and synchronous occurrence of CML and MCL has not been reported. Herein, we report the first case of synchronous CML and MCL in an otherwise healthy 38-year-old man. Potential etiologies and pathological relationships between the two malignancies are explored, including the possibility that the downstream effects of BCR-ABL may link it to an overexpression of cyclin D1, which is inherent to the etiology of MCL.

## 1. Introduction

Chronic myeloid leukemia (CML) and mantle cell lymphoma (MCL) are hematologic malignancies characterized by chromosomal translocations that are central to malignant transformation [1, 2]. The sequential occurrence of CML and MCL has been reported. A literature search yielded a case report of MCL which developed in a patient with CML treated with imatinib for three years and another case of blastoid variant of MCL in a patient of CML after achieving complete cytogenetic and molecular response to imatinib [3, 4]. Here, we report the synchronous development of CML and MCL.

## 2. Case Presentation

An otherwise healthy 38-year-old Caucasian male was found to have leukocytosis with a total white blood count (WBC) of 30.7 × 10^3^/mL during preoperative testing for an elective cervical spinal fusion and was referred for evaluation.

The patient worked as a sign hanger and was in his usual state of good health until he injured his neck several months prior. A month later, he was admitted with gastric ulcers and gastrointestinal (GI) bleed, both attributed to nonsteroidal anti-inflammatory drug use. His WBC during the admission was noted to be 15.3 × 10^3^/mL. The leukocytosis was presumed to be reactive and related to the GI bleed.

At the time of initial hematology-oncology consultation in an outside facility (six months after the GI bleed), the patient had no constitutional symptoms or evidence of any infection. He had received a steroid injection for neck pain four months prior. His family history was significant only for a maternal grandmother with colorectal cancer.

Physical exam was unremarkable, without evidence of pallor, palpable lymphadenopathy, or hepatosplenomegaly. His complete blood count (CBC) revealed a WBC of 26 × 10^3^/mL with 54% neutrophils, 39.5% lymphocytes, 6.5% monocytes, with hemoglobin (Hb) of 15.2 g/dL and a platelet count (PLT) of 199,000. His comprehensive metabolic panel was normal. Computerized tomography (CT) of his chest, abdomen, and pelvis showed no enlarged lymph nodes but revealed moderate splenomegaly (16.5 × 14.5 × 10 cm) and a nonspecific 6 mm pulmonary nodule in the right middle lobe.

Peripheral blood flow cytometry revealed a population of clonal B-cells with aberrant CD5 expression which was FMC7+, but neither CD20 nor lambda was dim, suggesting atypical chronic lymphocytic leukemia (CLL)/small lymphocytic lymphoma (SLL) or MCL. Fluorescence in situ hybridization (FISH) from the peripheral blood was positive for BCR-ABL/t(9;22) translocation, dual fusion (1R1G2F2A) 62%, consistent with a diagnosis of CML. FISH analysis also documented a small lymphocyte population (9.1%) containing CCDN1- IgH/t(11;14) and 17p-, indicating MCL. The patient was negative for JAK2 V617F and exon 12–14 mutations. He was referred to our center for a second opinion.

**Figure 1:**
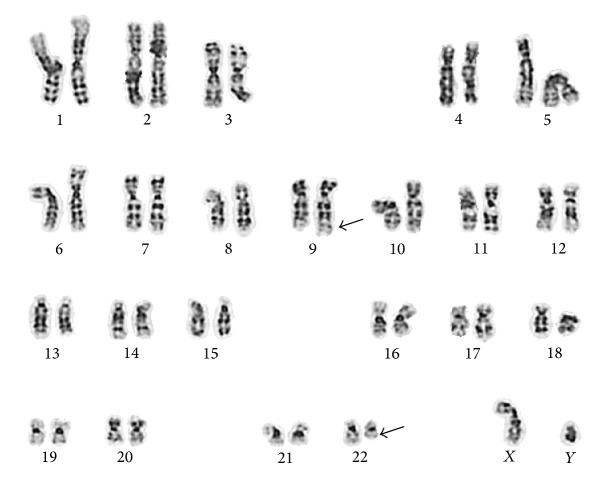
G-banded metaphase cell with 9/22 translocation (arrows indicate derivative chromosome 9 and 22 products).

Bone marrow (BM) biopsy showed a hypercellular marrow (95–100%) with trilineage hematopoiesis, dysmegakaryopoiesis, and myeloid hyperplasia (M : E ratio > 20 : 1) with the presence of lambda restricted clonal B-cells (5.5% by flow), without definite bone marrow involvement by B-cell lymphoma. A BM aspirate sample was harvested and cultured following 24 and 48 hours of incubation periods in* Chang Marrow *medium* (Irvine Scientific)* to facilitate analysis by conventional cytogenetic techniques. Chromosome spreads were G-banded by standard trypsin-Giemsa banding (GTG) technique and a total twenty metaphase cells were microscopically analyzed. Imaging and karyotyping were performed via computer-imaging techniques. Bone marrow cytogenetics revealed 46,XY,t(9,22)(q34;q11.2) [19/20]/46,XY [1/20] (Figure 1).

**Figure 2:**
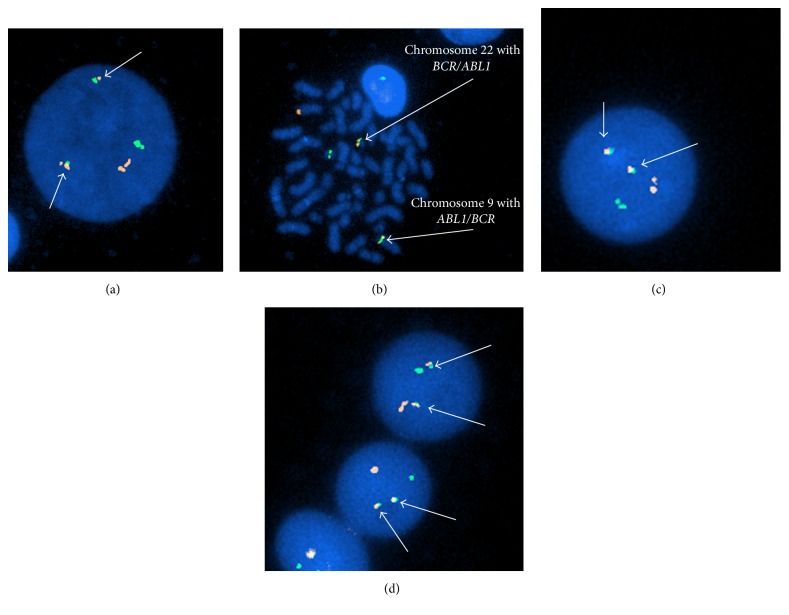
(a)* BCR*/*ABL1* dual fusion positive interphase nucleus; (b)* BCR*/*ABL 1 and ABL/BCR *positive metaphase cell (arrows indicate fusion products); (c) and (d)* IgH*/*CCND1* dual fusion positive interphase nuclei (arrows indicate fusion products).

Interphase FISH was positive for a BCR/ABL1 fusion rearrangement [p210 fusion protein by Quantitative Reverse Transcriptase Polymerase Chain Reaction (RT-qPCR)] and an IgH/CCND1 fusion rearrangement (dual fusion). The t(9,22) BCR/ABL1 fusion was identified in 88.3% cells by interphase FISH and the IgH/CCND1 fusion rearrangement was detected by interphase FISH in 4.7% cells. The identification of* BCR/ABL1* and* IgH/CCND1* gene loci fusion rearrangements were performed utilizing dual-color fusion DNA probe sets following the standard procedure as outlined by the probe manufacturer* (Abbott Molecular, Laboratories)*. Chromosome/nuclei counterstaining was performed by DAPI staining and a total of three hundred metaphase cells were microscopically analyzed for each probe set employed. Dual-color FISH images were digitally generated utilizing computer-imaging software (Figures 2(a)–2(d)).

The patient was started on Dasatinib for CML, and within a month his WBC normalized. After seven months of therapy, his spleen size on CT had decreased in size to 15 × 11 × 5.8 cm. Because he was asymptomatic without lymphadenopathy, his MCL was observed closely by serial peripheral blood flow analysis. At 10 months of follow-up, a repeat bone marrow evaluation showed acellular marrow specimen (50–60%) with trilineage hematopoiesis and normal maturation. The specimen also continued to contain a clonal population of B-cells (13%) consistent with MCL. Cytogenetic analysis revealed 46, XY (20) with interphase FISH negative for BCR/ABL1 gene loci fusion rearrangement and remained positive for IgH/CCND1 fusion rearrangement (4.3% of cells examined). Upper endoscopy and colonoscopy were performed and were negative for involvement with MCL. These results and results from RT-qPCR (BCRABL1/ABL1 ratio 0.0001) indicated that he attained a complete hematological and molecular response of his CML but had persistent though clinically asymptomatic MCL.

At 24 months of follow-up after initiation of treatment with Dasatinib, his CML remains in complete hematological and molecular remission. The MCL clone has persisted but remains stable, without development of measurable disease on symptoms. His MCL clone is being followed by serial peripheral blood flow cytometry, and at 24 months it demonstrates a waxing and waning (4–13%) small clonal B-cell population.

## 3. Discussion

The genetic alteration in CML is one of the most extensively studied and best understood models in human cancer, and the treatment of CML with tyrosine kinase inhibitors has been one of the most successful stories in oncology. From the first identification of the characteristic small chromosome in neoplastic cells from patients with CML by Nowell and Hungerford in 1960s [5], to the elucidation of the translocation and subsequent cytogenetic changes, we have come a long way understanding the biology of CML. The translocation t(9;22)(q34;q11) moves the ABL gene from the long arm of chromosome 9 to a small region BCR (breakpoint cluster region) producing a fusion oncogene BCR-ABL [6]. The BCR/ABL protein through its increased kinase activity is involved in neoplastic transformation via stimulation of multiple downstream signaling cross-connections. Some of the multiple pathways signaled by the BCR/ABL kinase are JAK/STAT, PI3K/AKT, and Ras/MEK, and these pathways drive CML pathogenesis by increasing cell proliferation; survival; decreasing apoptosis; cell differentiation; and altered cell adhesion [7].

The biology of CML involves a chronic phase with anemia, fatigue, splenomegaly with concomitant abdominal discomfort and infections which in a matter of 3–5 years progress into an accelerated phase ultimately leading to a blast crisis. There has been evidence that the hematopoietic (leukemic) stem cell with the BCR/ABL translocation can differentiate into either the common myeloid progenitors or the common lymphoid progenitors [8]. The evolution of accelerated phase and blast crisis is not fully understood but is presumed due to accumulation of more stem cell-like characteristics, increased beta-catenin activity, and additional mutations along the way, namely, Ph duplication, trisomy 8/19, and loss of 17p [9].

The blast crisis in CML may occur in either myeloid or lymphoid cell lines including extramedullary disease, and CML has been associated with non-Hodgkin lymphomas (NHL) in the setting of blast crisis [10]. In our patient, however, the appearance of MCL does not appear to be related to the myeloid etiology of CML, especially since he did not have accelerated phase or blast crisis at diagnosis.

MCL is a relatively infrequent B-cell lymphoma accounting for between 3 and 10% of NHL cases [11]. The t(11;14)(q13;q32) translocation places the Bcl-1 (B-cell lymphoma/leukemia) region next to the IgH (immunoglobulin heavy-chain) region on chromosome 14 causing an overexpression of cyclin D1 (CCND1). Cyclin D1 is a cell cycle protein which enables the G1/S phase transition, causing progression into the cell cycle [12]. Cyclin D1 achieves this effect by binding to cyclin dependent kinases (CDK) 4 and CDK6 forming a CDK/cyclin complex able to phosphorylate the retinoblastoma (RB1) gene- with subsequent release of the E2F transcription factor activating essential genes needed for S phase entry [13]. MCL cells are positive for the B-cell markers CD19, CD20, CD22, CD79a, and BSAP/PAX5. These malignant cells are also CD5+, FMC7+, Bcl-2-, Bcl-6-, and CD10-, with occasional weak positivity for CD23.

MCL is primarily diagnosed in the elderly and is relatively resistant to standard therapy with a median survival of 4-5 years [14]. These patients generally present with a variable clinical disease course, ranging from indolent to aggressive; at an advanced stage; and characterized by widespread lymphadenopathy, splenomegaly, and bone marrow involvement. Extranodal involvement and asymptomatic GI involvement is common while CNS involvement is noted in 10–20% of patients [13]. A study comparing the expression of BCR-ABL levels in bone marrow cells found a significantly higher number of BCR-ABL transcripts in association with higher expression of cyclin D1 levels in the accelerated phase (AP) of CML than in the chronic phase (CP) [15]. Another study showed that the activated ABL oncogene could signal through cyclin D1 and induce malignant transformation [16]. It is also suggested that one of the possible mechanisms involved in blast crisis could result from the accumulation of genetic and/or epigenetic changes including changes in the activity of the cyclin D/Rb pathway.

It is possible that through some of the above mechanisms attributed to BCR-ABL that cyclin D1 can be overexpressed to drive B-cell lymphomagenesis and potentially account for a possible biological link between CML and MCL. However, we do not believe this to be the case in our patient who developed both CML and MCL synchronously. Further, he responded completely to the TKI treatment specific for CML which had no effect on his stable MCL disease status. It seems more likely that two independent oncogenic processes developed in his bone marrow stem cells that led to the development of two independent malignancies. The eradication of the CML clone and the apparent stability of MCL over time also suggest that the MCL is a separate malignancy, biologically unrelated to the CML.

To our knowledge, we report the first case in which these two malignancies of different hematologic lineages appear to develop synchronously. Although there is no clear pathogenic link between the two malignancies, it is our conclusion that these two malignancies likely evolved from separate hematopoietic stem cells and further studies may be required to elucidate an association between CML and MCL.
